# Oral microbial signatures associated with age and frailty in Canadian adults

**DOI:** 10.1038/s41598-024-60409-8

**Published:** 2024-04-27

**Authors:** Vanessa DeClercq, Robyn J. Wright, Jacob T. Nearing, Morgan G. I. Langille

**Affiliations:** 1 Department of Pharmacology, Dalhousie University, Halifax, Nova Scotia Canada; 2 Department of Community Health and Epidemiology, Dalhousie University, Halifax, Nova Scotia Canada; 3 Department of Microbiology and Immunology, Dalhousie University, Halifax, Nova Scotia Canada

**Keywords:** Microbiome, Biomarkers, Risk factors, Epidemiology

## Abstract

This study aimed to assess the association between the oral microbiome, age, and frailty. Data and saliva samples were obtained from male and female participants aged 35–70 years (n = 1357). Saliva samples were analysed by 16S rRNA gene sequencing and differences in microbial diversity and community compositions were examined in relation to chronological age and the frailty index (FI). Most alpha diversity measures (Richness, Shannon Diversity, Faith’s Phylogenetic Diversity) showed an inverse association with frailty, whereas a positive association was observed with age and Shannon Diversity and Evenness. A further sex-stratified analysis revealed differences in measures of microbial diversity and composition. Multiple genera were detected as significantly differentially abundant with increasing frailty and age by at least two methods. With age, the relative abundance of *Veillonella* was reduced in both males and females, whereas increases in *Corynebacterium* appeared specific to males and *Aggregatibacter, Fusobacterium, Neisseria*, *Stomatobaculum,* and *Porphyromonas* specific to females. Beta diversity was significantly associated with multiple mental health components of the FI. This study shows age and frailty are differentially associated with measures of microbial diversity and composition, suggesting the oral microbiome may be a useful indicator of increased risk of frailty or a potential target for improving health in ageing adults.

## Introduction

Ageing is characterized by a wide range of physiological changes that impact human health as well as microbial populations on and within the human body. These microbes play key roles in the digestion and absorption of nutrients, production of metabolites, and the development and maintenance of intestinal, brain, and immune function^1–4^. Moreover, changes in the diversity and composition of these microbial populations can alter these critical functions and impact human health and disease.

In most studies of the gut microbiome that include adults (20–100 + years) from countries around the world, alpha diversity is higher with age and in long-lived groups (90 plus years)^5^. It has also been documented that beta diversity of the gut microbiome clusters by age group as well as living status (community-based vs long-term care residents)^5–9^. Importantly, chronological age itself is not the only age-associated contributor to microbial diversity, there is marked variation in the rate of biological aging, and accumulating evidence suggests the microbiome may be reflective of this. For example, Wilmanski et al. examined three independent cohorts across the US, looking at over 9000 individuals aged 18–101 years and showed strong positive association between chronological age and beta diversity of the gut, however, microbial composition was altered when stratified by health status^10^. Frailty is a reduction in health and functioning accompanying ageing and is characterized by increased vulnerability to adverse health outcomes. The frailty index (FI) can be used as a model of biological ageing^11,12^, reflecting the cumulative deterioration of multiple physiological and psychological systems. Multiple studies have demonstrated that higher levels of frailty correlate with reduced gut alpha diversity^13–17^, significant influences on beta diversity^13,17–19^, and changes in composition of the gut microbiome^13–22^.

Each location on the human body represents a distinct niche with varying degrees of microbial diversity and unique community structures^23–25^. For instance, the gut microbiome is dominated by the genera *Bacteroides, Prevotella, Ruminococcus*, and *Blautia* whereas the oral cavity is dominated by the genera *Streptococcus, Neisseria, Prevotella, Veillonella*, and *Haemophilus*^24,26–28^. Although research on age and the oral microbiome is not as extensive as the gut, there have been some studies that have compared microbial diversity in different oral sites. For example, reduced alpha diversity has been observed in gingival crevicular fluid, the tongue, and saliva with increasing age,^29,30^ but was similar across ages in subgingival plaque^31^. Shifts in beta diversity with age have also been documented in different oral sites^29–31^. Additionally, a small study comparing salivary microbiomes of individuals living in nursing homes (68–101 years old; n = 15) to those living independently (79–94 years old; n = 16) showed distinct classification between the two groups using unsupervised principal component analysis as well as significant differences in specific taxa^32^ suggesting that there is an association between oral microbiota composition and frailty. More recently, a study in predominantly females from the TwinsUK cohort showed an inverse association between frailty and saliva microbiota alpha diversity^33^.

With a lack of research on the oral microbiome and frailty, we sought out to investigate the association between salivary microbiota and both frailty and chronological age in community living male and female adults. To address this aim, data and samples were accessed from the Atlantic Partnership for Tomorrow’s Health (PATH) cohort. This is a large population cohort (age 30–74 years) in the Atlantic region of Canada which includes detailed questionnaire data, biological samples, and clinical measures^34,35^. A portion of participants also have FI data^36^ and 16S rRNA amplicon sequencing data from saliva samples^28,37,38^.

## Results

### Characteristics of participants

To assess the association between both chronological age and biological age with oral microbiome diversity and composition, only participants that had age, frailty and oral microbiome sequencing data available were included (n = 1357). Saliva samples were sequenced using 16S rRNA gene sequencing of the V4-V5 region, with median sequencing depth of 18,668 reads per sample after processing (range 2974–120,017; n = 1795), and were assigned taxonomy using the expanded Human Oral Microbiome Database^39^. The median age of the participants was 57 years, and the median FI score was 0.13 (Table 1). The median FI score indicates that the participants were relatively healthy, with the majority being considered robust or scoring below pre-frailty^40,41^. Considering that the oral microbiome varies by sex^28^ and that frailty tends to be higher in females than males^42,43^, a sex-stratified analysis was also conducted. The characteristics of participants by sex are presented in Table 1. There were a higher proportion of female than male participants, and the female participants had a higher FI, but lower chronological age, body weight, height and BMI. The majority of participants reported using at least 1 medication, were non-smokers, consumed 2 servings of vegetables per day, and had a BMI in the overweight range.

**Table 1:** Characteristics of participants by sex.

	Overall	Males	Females
Sex—M/F			
n [%]	446/911 [33/67]	446/0 [100/0]	0/911 [0/100]
Age			
Median [IQR]	57 [48–60]	59 [51–61]	56 [47–59]
Frailty			
Median [IQR]	0.13 [0.08–0.18]	0.12 [0.08–0.18]	0.13 [0.08–0.19]
Weight (kg)			
Median [IQR]	76 [66–87]	86 [78–95]	70 [64–80]
Height (kg)			
Median [IQR]	167 [161–174]	177 [172–181]	163 [159–167]
BMI (kg/m^2^)			
Median [IQR]	27 [24–30]	28 [25–30]	27 [23–30]
Smokers count			
n [%]	43 [3]	15 [3]	38 [3]
Vegetable servings/d			
Median [IQR]	2 [1–3]	2 [1–3]	2 [2, 3]
Medication use, n [%]			
None	617 [44]	216 [46]	401 [30]
1 Medication	339 [25]	105 [24]	234 [26]
≥ 2 Medications	418 [30]	125 [28]	276 [30]

### Frailty and age show divergence in alpha diversity patterns

**Figure 1:**
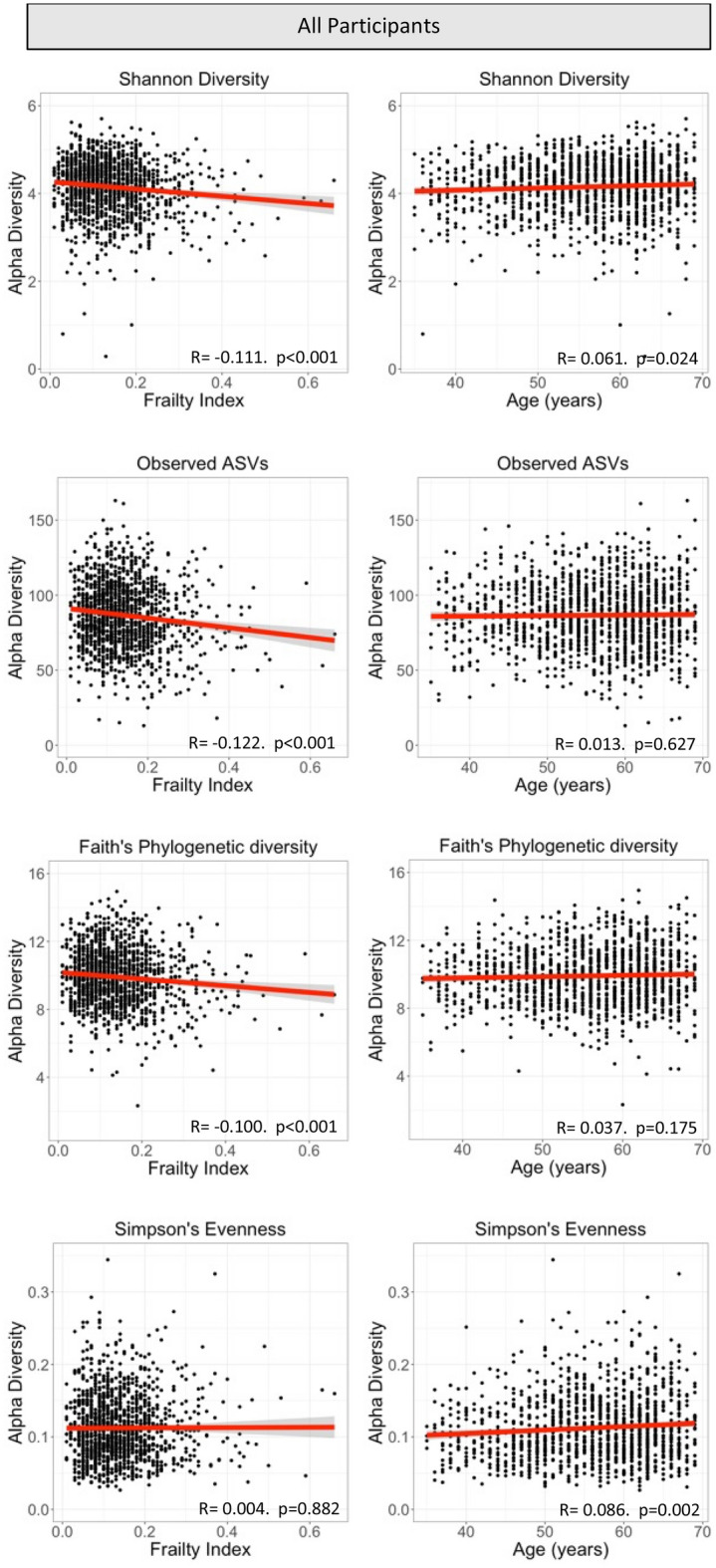
Correlation between alpha diversity, frailty, and age in all participants. Alpha diversity is represented by Shannon diversity, Observed ASVs (richness), Faith’s phylogenetic diversity, and Simpson’s evenness. Regression coefficients and p-values are obtained from Pearson’s correlation.

**Figure 2:**
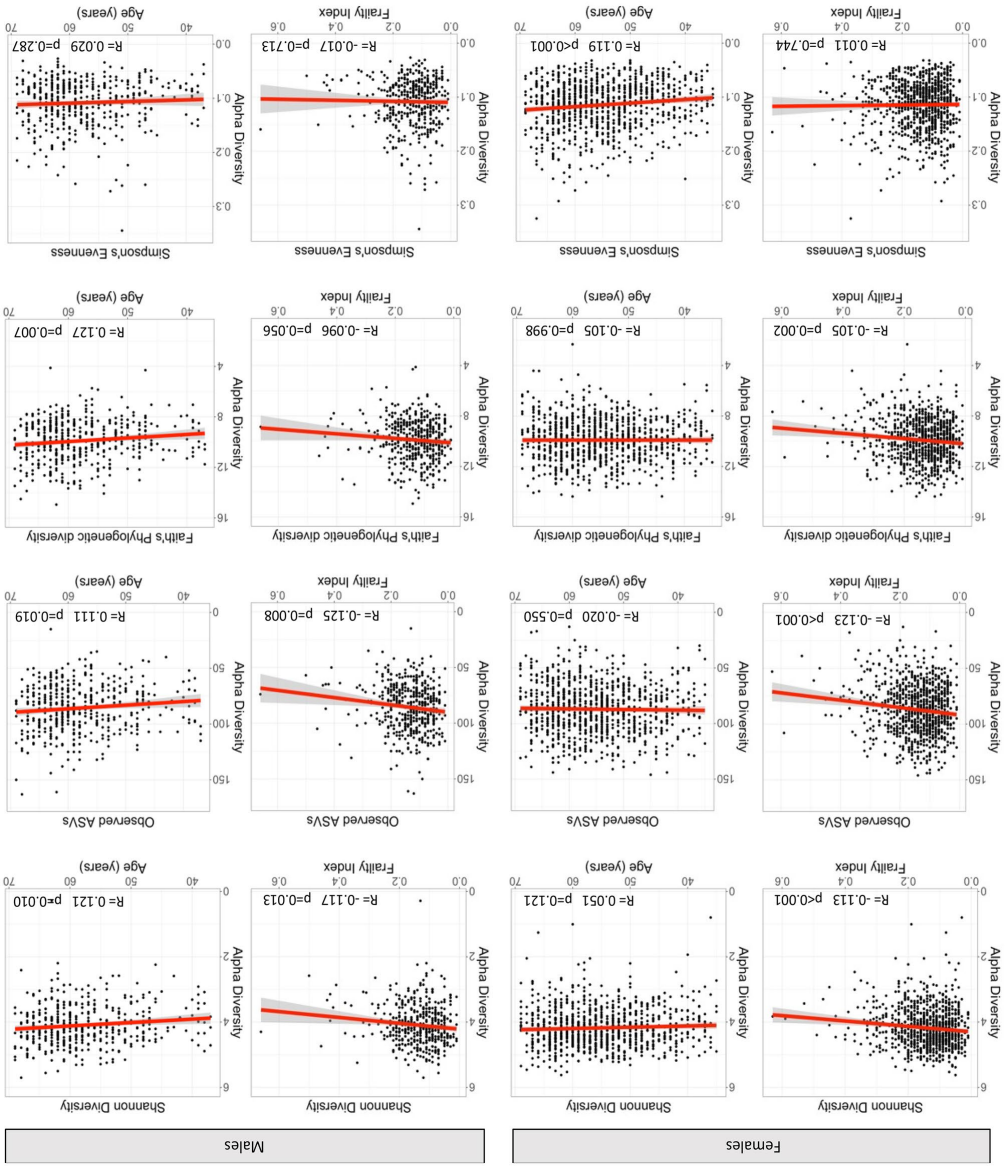
Correlation between alpha diversity, frailty, and age in male and female participants. Alpha diversity is represented by Shannon diversity, Observed ASVs (richness), Faith’s phylogenetic diversity, and Simpson’s evenness. Regression coefficients and p-values are obtained from Pearson’s correlation.

As measured by Pearson correlation, all measures of alpha diversity showed significant inverse correlation with frailty (p < 0.001), except evenness (Fig. 1). On the other hand, Evenness (p = 0.002) and Shannon Diversity (p = 0.024) were positively correlated with age, but Observed ASVs (richness) and Faith’s Phylogenetic Diversity were not associated with age (Fig. 1). In the sex-stratified analysis, high frailty was inversely correlated with Shannon Diversity (p < 0.001), Observed ASVs (p < 0.001), and Faith’s Phylogenetic Diversity (p = 0.002), but not evenness in females (Fig. 2). Increasing age positively correlated with evenness (p < 0.001), but none of the other alpha diversity measures in females (Fig. 2). In contrast, increasing age positively correlated with all measures (p < 0.02) except evenness in males (Fig. 2). In males, increasing frailty showed a similar pattern to females with a significant inverse correlation with Shannon Diversity (p = 0.013), and Observed ASVs (p = 0.008), and a trend with Faith’s Phylogenetic Diversity (p = 0.056) (Fig. 2).

### Beta diversity is associated with frailty and age

**Figure 3:**
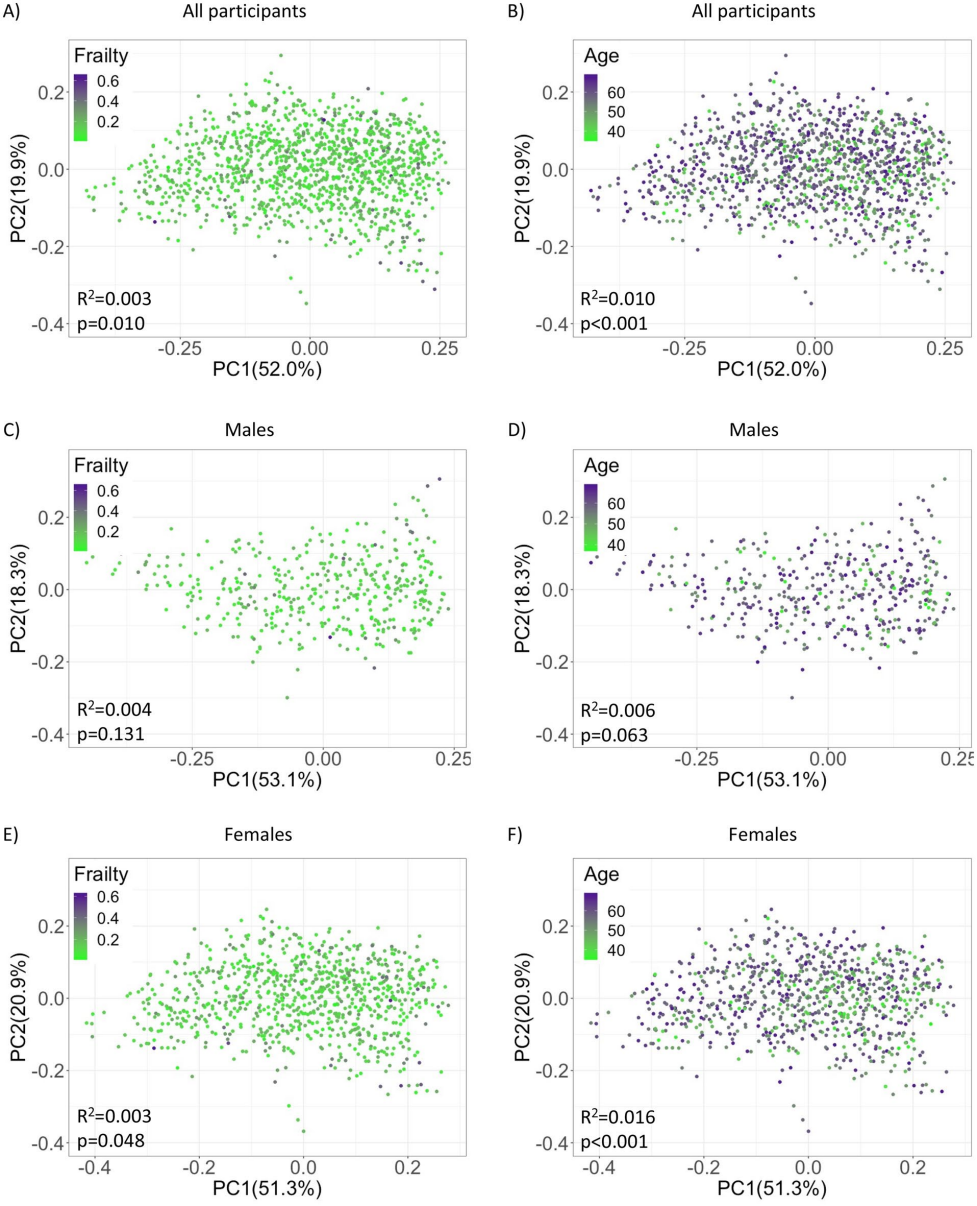
Beta diversity analyses among frailty and age groups are represented by Principal Coordinates Analysis plots based on weighted UniFrac. (**A**,**B**) all participants, (**C**,**D**) male participants, (**E**,**F**) female participants. R^2^ and p-values are the result of the unadjusted PERMANOVA test.

The association between beta diversity and age and frailty was using a PERMANOVA and assessed using three different metrics, weighted UniFrac distance, Bray–Curtis dissimilarity, and Robust Aitchison’s distance. A significant association was found between frailty and the beta diversity measures weighted UniFrac distance and Bray–Curtis dissimilarity in the unadjusted analysis (Fig. 3a and Supplementary Fig. 1a) but did not remain significant in the model adjusted for sex, smoking status, height, weight, vegetable consumption, and medication use (weighted UniFrac distance R^2^ = 0.001, p = 0.184; Bray–Curtis dissimilarity R^2^ = 0.001, p = 0.396). In contrast, a significant association was found between age and all 3 beta diversity measures in both the unadjusted (Fig. 3b, Supplementary Figs. 1, and 2) and adjusted models (weighted UniFrac distance R^2^ = 0.013, p < 0.001; Bray–Curtis dissimilarity R^2^ = 0.008, p < 0.001; Robust Aitchison’s distance R^2^ = 0.004, p < 0.001).

Further analysis in males only, showed that none of the beta diversity measures were significantly associated with frailty in the unadjusted (Fig. 3c, Supplementary Figs. 1c and 2c) or adjusted models (weighted UniFrac distance R^2^ = 0.002, p = 0.350; Bray–Curtis dissimilarity R^2^ = 0.003, p = 0.133) whereas with age, a trend (weighted UniFrac and Bray–Curtis) and significant (Robust Aitchison’s distance) association was observed with the beta diversity measures in the unadjusted models (Fig. 3d, Supplementary Figs. 1d and 2d) and a significant association with all measures in the adjusted model (weighted UniFrac R^2^ = 0.006, p = 0.041; Bray–Curtis dissimilarity R^2^ = 0.005, p = 0.039; Robust Aitchison’s distance R^2^ = 0.005, p = 0.007).

In females, the beta diversity measures weighted UniFrac distance and Bray–Curtis dissimilarity were significantly associated with frailty in the unadjusted (Fig. 3e and Supplementary Fig. 1e) but not in the adjusted models (weighted UniFrac distance R^2^ = 0.001, p = 0.504; Bray–Curtis dissimilarity R^2^ = 0.001, p = 0.556), whereas with age in females, a significant association was found with all beta diversity measures in both the unadjusted (Fig. 3f, Supplementary Figs. 1f and 2f) and adjusted models (weighted UniFrac distance R^2^ = 0.016, p < 0.001; Bray–Curtis dissimilarity R^2^ = 0.011, p < 0.001; Robust Aitchison’s distance n R^2^ = 0.004, p = 0.003). In recognition of the smaller sample size of males in our study, a separate analysis was conducted in a subset of females that were down sampled to the same sample size as the male group (n = 446) and matched on age (± 2 year). In this smaller subset of females, some of the effect size is lost but all beta diversity measures remain significantly associated with age in the unadjusted models (Supplementary Fig. 3) and remain significant in the adjusted models (weighted UniFrac distance R^2^ = 0.007, p = 0.031; Bray–Curtis dissimilarity R^2^ = 0.006, p = 0.011; Robust Aitchison’s distance R^2^ = 0.005, p = 0.017). However, with frailty, statistical significance is lost in the unadjusted (Supplementary Fig. 4) and adjusted models (weighted UniFrac distance R^2^ = 0.001, p = 0.726; Bray–Curtis dissimilarity R^2^ = 0.002, p = 0.637; Robust Aitchison’s distance R^2^ = 0.002, p = 0.666). These results suggest that some statistical power may be lost with smaller samples sizes, especially with highly skewed variables with long tails such as frailty, but the smaller sample size is sufficient for observing associations in variables with shorter-tailed distributions such as age (Supplementary Fig. 5).

### Taxonomic composition differs with frailty and age

We used four different differential abundance (DA) tools to identify genera or ASVs that might be associated with frailty and age. With increasing frailty, we found 25 genera that were associated with frailty in the unadjusted model (Table 2). At the genus level, *Alloprevotella*, *Leptotrichia, Peptococcus, Selenomonas,* and unclassified members of the *Lachnospiraceae* and *Ruminococcacceae* families were detected as significantly lower with increasing frailty, whereas *Rothia, Streptoccus,* and *Veillonella* were significantly increased with frailty by two or more DA tools in the unadjusted model (Table 2). In the adjusted model, only *Lachnospiraceae* remained statistically significant by two or more DA tools (Table 2). At the genus level, both *Alloprevotella* and *Veillonella* decreased with increasing age, whereas *Abiotrophia, Aggregatibacter, Capnocytophaga, Neisseria, Porphyromonas, Stomatobaculum, Tannerella, Treponema,* and unclassified members of the Peptostreptococcaceae family and *Bacteroidales* order were detected as significantly increased with age by two or more DA tools in the unadjusted model, and most remained significant in the adjusted model (Table 3).

**Table 2:**
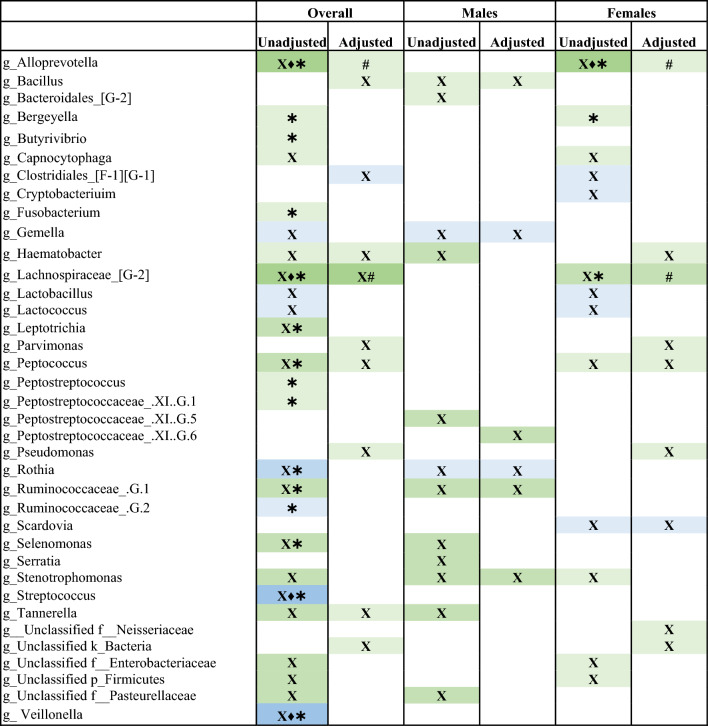
Genera detected as significantly different with increasing frailty.

Four different differential abundance tools were used to detected genera significantly associated with frailty. Differential abundance tools: Corncob (X), ANCOM-II (♦), MaAsLin2 (✱), and ALDEx2 (#).

Blue indicates an increase in relative abundance with increasing frailty; green indicates a decrease in relative abundance with increasing frailty. Increasing colour intensity of a cell indicates a great number of tools that identified a genus as differentially abundance.

**Table 3:**
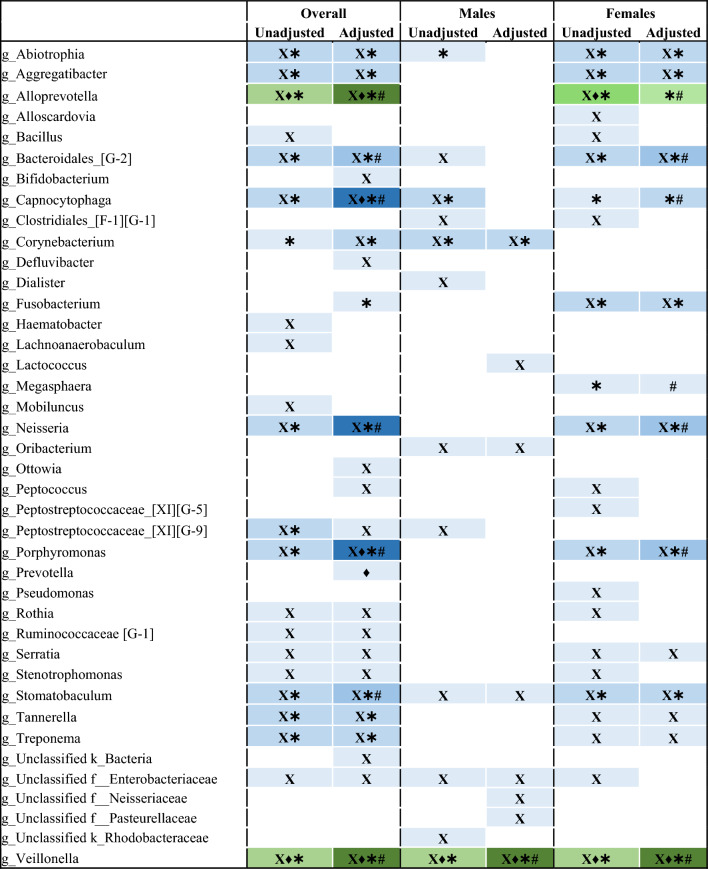
Genera detected as significantly different with increasing age.

Four different differential abundance tools were used to detected genera significantly associated with chronological age. Differential abundance tools: Corncob (X), ANCOM-II (♦), MaAsLin2 (✱), and ALDEx2 (#).

Blue indicates an increase in relative abundance with increasing age; green indicates a decrease in relative abundance with increasing age. Increasing colour intensity of a cell indicates a great number of tools that identified a genus as differentially abundance.

In the sex-stratified analysis, we found several genera that were associated with increasing frailty and age, and many of these differed between males and females. With increasing frailty in males, no genera were detected as significantly different by at least two DA tools, but in females *Alloprevotella* and a member of the *Lachnospiraceae* family were significantly lower with increasing frailty in the unadjusted model (Table 2). In males, *Capnocytophaga* and *Corynebacterium* were significantly increased while *Veillonella* was significantly lower with increasing age by at least two DA tools in the unadjusted model (Table 3), and associations with *Corynebacterium* and *Veillonella* remained significant after adjusting for covariates (Table 3). With increasing age in females, 9 genera were detected as significantly different in females by at least two DA tools in the unadjusted model and all associations remained significant after adjusting for covariates (Table 3), including a significant a decrease in *Alloprevotella and Veillonella,* and an increase in *Abiotrophia, Aggregatibacter, Capnocytophaga, Fusobacterium, Neisseria, Porphyromonas, Stomatobaculum,* and an unclassified genus of the *Bacteroidales* order (Table 3). With increasing age in males, 3 genera were detected as significantly different in males by at least two DA tools in the unadjusted model, and after adjusting for covariates a significant decrease in *Veillonella* and an increase in *Corynebacterium* remained (Table 3).

### Contribution of individual frailty components

We examined the relationship between beta diversity and the 38 different variables that were part of the Atlantic PATH FI^36^, including questions on mental health, self-reported diagnosed chronic conditions and health, and physical health measures. We found that 3 of the 16 FI variables associated with mental health were detected as significant with all 3 beta diversity measures, and 5 of the 16 FI variables with at least 2 measures. Only 1 of the 10 physical measurements (fat free mass) was significant with all beta diversity metrics, and 1 of the 10 self-reported diagnoses (osteoporosis) was detected as significant with two beta diversity metrics (Table 4).

**Table 4:** Frailty Index components associated with beta diversity.

Instrument	Variable	Weighted UniFrac		Bray–Curtis		Robust Aitchison	
		R^2^	p-value	R^2^	p-value	R^2^	p-value
Atlantic PATH questionnaire	Avoid food mouth problem	0.002	0.101	0.002	0.055	**0.002**	**0.037**
CPTP core questionnaire	Self-rated general health	0.002	0.080	**0.002**	**0.041**	0.001	0.205
Patient health questionnaire-9 (PHQ-9)	Little interest	**0.004**	**0.010**	**0.002**	**0.019**	**0.002**	**0.049**
	Depressed	0.002	0.092	0.002	0.100	0.001	0.083
	Sleep issues	0.001	0.503	0.001	0.338	0.001	0.438
	Tired	**0.004**	**0.012**	**0.002**	**0.019**	**0.002**	**0.028**
	Eating problem	**0.003**	**0.021**	**0.002**	**0.041**	0.001	0.154
	Self-confidence problem	0.001	0.554	0.001	0.604	0.001	0.595
	Concentration issues	**0.003**	**0.042**	0.001	0.118	0.001	0.345
	Slow fast problems	0.002	0.074	0.002	0.055	0.001	0.206
	Suicidal	0.002	0.096	**0.002**	**0.050**	0.002	0.062
General anxiety disorder-7 (GAD-7)	Nervous	0.002	0.096	0.001	0.559	0.001	0.364
	Uncontrolled worrying	**0.003**	**0.036**	0.001	0.306	0.001	0.229
	Chronic worrying	0.002	0.073	0.001	0.363	0.001	0.231
	Trouble relaxing	**0.003**	**0.022**	0.002	0.092	0.001	0.155
	Restless	**0.005**	**0.005**	**0.002**	**0.014**	0.001	0.120
	Easily annoyed	**0.005**	**0.001**	**0.003**	**0.012**	**0.002**	**0.037**
	Feeling afraid	0.002	0.069	0.001	0.123	0.001	0.364
Diagnosed conditions	HBP	0.001	0.660	0.001	0.522	0.001	0.205
	MI	0.001	0.211	0.002	0.107	0.001	0.760
	Stroke	0.001	0.385	0.001	0.340	0.0001	0.173
	CB	0.002	0.100	0.002	0.080	**0.002**	**0.015**
	COPD	0.002	0.113	0.001	0.312	**0.002**	**0.023**
	DM	0.001	0.636	0.001	0.663	0.001	0.829
	IBS	0.001	0.544	0.001	0.384	0.002	0.075
	Osteoporosis	**0.003**	**0.035**	**0.002**	**0.024**	0.001	0.106
	Arthritis	0.001	0.251	0.001	0.207	0.001	0.288
	Cancer	0.001	0.317	0.001	0.219	0.001	0.559
Physical health measurement	SBP	0.001	0.370	0.001	0.431	0.001	0.749
	DBP	0.001	0.414	0.001	0.410	0.001	0.126
	HR	0.002	0.086	0.002	0.060	0.001	0.106
	PP	0.001	0.400	0.001	0.296	0.001	0.372
	WC	0.001	0.193	**0.002**	**0.033**	0.002	0.075
	FM	0.001	0.313	0.000	0.628	0.001	0.740
	FFM	**0.005**	**0.005**	**0.004**	**0.002**	**0.002**	**0.012**
	BMR	0..000	0.904	0.001	0.881	0.001	0.700
	BMI	0.001	0.454	**0.002**	**0.029**	0.001	0.139
	Grip	0.000	0.891	0.001	0.627	0.001	0.610

Bold indicates statistically significant associations with beta diversity as measured by weighted UniFrac, Bray–Curtis Dissimilarity, or Robust Aitchison Distance.

## Discussion

To date the research on the oral microbiome and frailty is limited to a few studies in select populations, often with very small sample sizes and a lack of consideration for sex specific responses. Thus, the current study added to our growing body of knowledge by examining the sex-specific relationships between oral microbiomes, ageing and frailty in a large population cohort of Canadians. Using 16S rRNA gene sequencing data from saliva samples, we showed that microbial diversity and composition differ by sex and with increasing frailty and chronological age. In the overall cohort, most alpha diversity measures declined with increasing frailty, in contrast alpha diversity increased or remained unchanged with increasing age. More specifically an inverse relationship between microbial richness and frailty was observed in both males and females, whereas a positive relationship between microbial richness and age were observed in males only. A significant association was noted between beta diversity and age overall, as well as in the sex-stratified analysis even after adjusting for covariates. This association only remained significant in females. Finally, using multiple DA tools we demonstrated that several taxa were either increased or decreased with frailty and age. These findings expand the current state of knowledge on the oral microbiota with frailty and age in males and females.

Much of the previous microbiome literature on ageing has been conducted on the gut microbiota. For instance, in 2020 Badal et al. conducted a large systematic review on 27 studies from around the globe on adults 20–100 + years of age to assess ageing-associated changes in the gut microbiome^5^. The systematic review showed an overall higher alpha diversity with age and in long-lived groups (90 plus years) and significant differences in beta diversity between age groups^5^. The findings of the 2020 systematic review were supported by more recent research showing similar trends with age and both alpha and beta diversity of the gut^6–9^.

In agreement with the current findings of an increase in Shannon diversity with age and no change in richness (Fig. 1), Well et al. also reported a positive association between age and Shannon diversity and a non-significant association with richness of the salivary microbiome (n = 679) in members of the TwinsUK cohort^33^. In contrast, Schwartz et al. showed a decrease in richness (Chao1) and Shannon diversity of the salivary microbiome with age in adults from the US (n = 271)^30^. However, the study by Schwartz et al. did not consider general overall health or frailty of the participants, whereas when we examined our participants by frailty a clear decline in multiple measures of alpha diversity was observed (Fig. 1). Indicating that aging and frailty have differential effects on the diversity of the oral microbiome. Moreover, alpha diversity of the oral microbiome may also vary by location within the oral cavity. For example, we demonstrated a slight increase in Shannon diversity of the salivary microbiome with age (Fig. 1), whereas Larson et al. showed a decrease in the tongue dorsum microbiome in community dwelling adults^44^, highlighting that differing oral communities may potentially respond differentially to ageing.

In the current study beta diversity of the salivary microbiome was significantly associated with age in the overall cohort (Fig. 3, Supplementary Figs. 1 and 2), which is in agreement with the studies by Wells et al.^33^ and Schwartz et al.^30^. Further analysis in these studies demonstrated that in addition to age, the following covariates contributed significantly to microbial variation: dentate, tobacco use, active caries, periodontal status and, gender/sex, whereas BMI and medication used failed to show a significant association with variation. Similarly, our team has previously shown minimal variation due to medication use within the Atlantic PATH cohort^38^, and only small associations between BMI and select beta diversity measures^28^. Tobacco use (Table 1) was very rare (3%) in the current cohort and included in the adjusted models. Furthermore, in a sensitivity analysis where currently daily smokers were removed, the associations with beta diversity metrics remained consistent, showing significant associations with age in both the unadjusted and adjusted models (Supplementary Fig. 6). Unfortunately, the Atlantic PATH cohort did not collect detailed oral health information related to dentate, gingival bleeding, active caries, or periodontal status. The only oral health information collected was time since last dental visit, and this variable was explored previously, but it was only associated with one beta diversity measure (Bray–Curtis dissimilarity) and unrecoverable within the leave-one-out cross-validation cohort^28^. On the other hand, unlike most previously published studies, our study of the salivary microbiome was large enough to allow for a sex-stratified analysis, which revealed that the significant association between age and beta diversity on both males and females in the adjusted models (Fig. 3, Supplementary Figs. 1 and 2), and remained statistically significant in a smaller subset females matched to males (Supplementary Fig. 3).

Moving beyond chronological age, recent research has started to shed light on the relationship between the oral microbiome and frailty. For example, Wells et al.^33^ examined how frailty and diet influence the salivary microbiome in members of the TwinsUK cohort (n = 679), demonstrating a significant association between frailty and three alpha diversity measures^33^. These finding agree with the current study showing differences in multiple measures of alpha diversity with increasing degrees of frailty (Fig. 1). Both the current study and the Wells et al. study^33^ examined frailty using the same systematic process for the generation of the FI^40^. Taking another approach, Ogawa et al. examined the salivary microbiome of frail individuals living in nursing homes (n = 15, only 3 males) to healthy independent living (n = 16, 7 males) older individuals in Japan^32^. They found Shannon diversity at the phylum level was significantly lower in individuals living in a nursing home than independently living, and clear clustering (beta diversity) of individuals living in nursing homes compared to independently living^32^.

The observed divergence in oral microbiome results between age and frailty are not unique to the oral microbiome, differences with chronological age and frailty have also been noted in the gut microbiome^45^. This incongruity between age and frailty noted in both the current study and recent literature, likely reflects cumulative exposures to environmental and lifestyle factors as well as the physiological differences related to health status during the ageing process. Healthy individuals appear to have a relatively stable microbiome with a rich diversity that may reflect the almost consistent environmental challenges from mastication, saliva production, oral hygiene routines, and the hosts diet, which would not only bring a source of nutrients to the resident microbes but also the introduction of new microorganisms. On the other hand, frailty has been associated with reduced diversity in both the gut and oral cavity^13,18,19,33,45–48^. This reduction in microbial diversity may be reflected by complexity of frailty involving the deterioration of multiple physiological and psychological systems. For instance, many of the health deficits that contribute to frailty (e.g. chronic disease, physical strength/muscle mass, and mental health conditions) have been associated with reduce diversity^49–54^. Many of these conditions are characterized by chronic inflammation and compromised immune function, thus accumulated health deficits observed with frailty may potentially cause multiple shifts in the composition of microbiota depending on the systems affected. In addition, the influence of medications taken for multiple components of frailty may also directly or indirectly influence the composition or function of the oral microbiome (e.g. altering metabolic pathways or saliva production). Many biological and socio-behavioral factors could possibly attribute to differences in oral microbiome results between age and frailty. For instance, previous research has demonstrated sex differences in microbial diversity^28^ and a higher prevalence of frailty in females than males^42,43^, thus the higher number of females in this study may play a part in the age-frailty divergence. Additionally, gender related habits such as diet may result in different intakes of dietary pre- and pro-biotics and further differences in exercise and body composition (e.g. muscle mass) may be differentially influenced by microbial metabolites. Therefore, the underlying biological mechanisms responsible for the differing relationship between chronological age and frailty with the oral microbiome are complex, likely involve multiple mechanisms, and merit further investigation.

In the current study, we also identified several taxa that varied by age and frailty (Tables 2 and 3). The study by Schwartz et al. showed changes in several species with age, some belonging to the same genera that we noted in the current study such as *Porphyromonas,* and *Alloprevotella*^30^. Additionally, our findings are in agreement with Wells et al., demonstrating an inverse association between age and *Veillonella* abundance^33^. With respect to frailty, Ogawa et al. found significant differences in several genera between individuals living in nursing homes compared to independent living including *Veillonella*, *Capnocytophaga, Fusobacteriuim, Leptotrichia, Streptococcus, and Selenomonas* which is consistent with our findings with at two or more DA tools (Table 2). *Veillonella* are anaerobic gram-negative bacteria that ferment organic acids, such as lactate^55^, and may be reflective of oral hygiene and number of teeth^56–58^ and has been linked to increased cardiometabolic risk^59^.

Finally, we explored the influence of individual components of the FI on community composition of the salivary microbiome, showing an association with several of the mental health variables (Table 4). Although, oral microbiome research examining specific components of the FI is lacking, the gut microbiome has been explored. In contrast to the current oral microbiome study, Lim et al. examined specific measures of frailty with Bray–Curtis dissimilarity in the gut microbiome and reported a small but significant association with grip strength, and no association with BMI and waist circumference^16^. On the other hand, both the above-mentioned gut microbiome study and the current salivary microbiome study showed a nonsignificant association with blood pressure and a significant association between beta diversity and depression. Furthermore, recent oral microbiome research focused on young adults showed that the composition of the oral microbiome differed significantly between participants with depressive disorder and those with no history of mental health problems^60^.

As with any population-based study, we acknowledge our study has some limitations. One of the main limitations is the lack of information available on oral health, including details on dentate, caries, or gingival bleeding. Previous research has shown that dental calculus, frequency of gum bleeding, flossing, and brushing are all associated with salivary microbiome composition^61^. Likewise, oral health grades have been shown to be positively associated with alpha diversity measures (richness and Shannon index)^62^. Some of these covariates also showed sex-specific associations. For example, the frequency of gum bleeding explained a larger proportion of variation in salivary microbial composition in females (*n* = 2509) than males (*n* = 1955)^63^. Additionally, oral health problems have been associated with mental health disorders^51^, thus some of the microbiome signal observed with mental health components of the FI could be contributed by poor oral health. Another limitation is the lack of diversity and representativeness for some sociodemographic characteristics. For example, income and education levels are above average compared to Canadian census data^35^, however, our previous work indicates that these two variables do not contribute significantly to salivary microbial variation^28^. Lastly, the proportion of specific ethnic identities reported in the Atlantic PATH dataset were similar to Canadian census data, but numbers in traditionally underrepresented groups are too low to allow us to fully examine the influence of ethnicity on the oral microbiome. These limitations restrict our ability to identify discrete microbial signatures across diverse populations.

Our study also has several strengths, which greatly enhances the existing literature on the oral microbiome in the areas of frailty and ageing. To our knowledge, this is largest oral microbiome study to date to examine frailty, with sequencing data on nearly 1400 saliva samples. As such, the large sample size allowed us to conduct a sex-stratified analysis, providing sex-specific findings that were previously lacking in the literature. While some overall trends were observed with both sexes, distinct patterns with specific taxa were exposed. Also, the Atlantic PATH cohort shows overall congruence with Canadian Census data for the age groups of adults living in the Atlantic region^35^. In addition, previous research on the salivary microbiome and frailty has shown drastic divergence in beta diversity between community living individuals and those living in a nursing home^32^, thus using the Atlantic PATH cohort of all community living individuals to study frailty may minimize some confounders that could change in a nursing home setting, such as dietary intake and oral health routines. Finally, the cohort collected many covariates including information on smoking status, diet, anthropometric measures, and sociodemographic factors. We and others have previously examined multiple covariates for their contribution to microbial variation, showing that variables such as BMI and diet explained < 1% of the variation^28,33,61^, thus giving us confidence that such confounding variables minimally influence the composition of the oral microbial community. This was further verified in the current study, where the results were minimally influenced when adjusting for several of those variables.

## Conclusions

In conclusion, results from this study show age and frailty are differentially associated with measures of microbial diversity and composition of the salivary microbiome and further vary by sex. Using 16S rRNA gene sequencing data from saliva samples, we observed a decline in several alpha diversity measures and different clustering patterns with increasing degrees of frailty in community living Canadians who were of similar age. Our results suggest that overall frailty is one factor associated with oral microbiome diversity and composition and that the salivary microbiome may be a useful indicator of increased risk of frailty. Our findings also identify several taxa that were increased or decreased with frailty and age in a sex dependent manner. Although frailty is an accumulation of multiple factors, we found that many of the mental health components of our FI measure were associated with the oral microbiome. Future research should consider when the oral microbiome changes occur in relation to the development or degrees of frailty, as well as incorporate recruitment strategies that would capture a more diverse population and allow for analysis of samples from underrepresented populations. Finally, the oral microbiome may be a potential target for improving health and future studies are needed to elucidate the role of specific taxa as therapeutic options for ageing adults.

## Methods

### Ethics

This study has been conducted using Atlantic Partnership for Tomorrow’s Health (PATH) data and biosamples, under application #2018-103. This study was conducted in compliance with the guiding principles of the Declaration of Helsinki and was approved by the provincial and regional ethics committees in each Atlantic province (New Brunswick: Horizon Health Network and Vitalité Health Network; Nova Scotia: Nova Scotia Health Authority Research Ethics Board and IWK Research Ethics Board; Newfoundland and Labrador: Health Research Ethics Board Newfoundland; Prince Edward Island: Health Prince Edward Island). All Atlantic PATH participants provided written informed consent before participation in the study. Research ethics board approval for the use of secondary data and biological samples used in current study was granted by Dalhousie University.

### Study design and cohort

A cross-sectional study design was used to assess the association between the oral microbiome and age groups and degrees of frailty in a large population cohort. This study utilized data and samples from the Atlantic PATH cohort, a regional cohort of the Canadian Partnership for Tomorrow’s Health (CanPath) Project. Details on participant recruitment, data collection, and a cohort profile have been reported previously^34,35^. In short, participants were 30–74 years at the time of recruitment, completed a standardized set of questionnaires (available at: https://www.atlanticpath.ca/), which included questions on diet, smoking status, sex, age, and medication use. A subset of participants had anthropometric measures and biological samples, including saliva, collected. A portion of those participants had a calculated frailty score (n = 9133)^36^ and a nested subset of saliva samples had been previously analyzed by 16S rRNA amplicon sequencing (n = 1711)^28,37^. For the current study, male and female participants were included if they had available oral microbiota sequencing and frailty data (n = 1357).

### Oral sample collection

Stimulated saliva samples were collected during normal clinic hours (9:00 a.m.–7:00 p.m.), after completing an approximately 1-h interview and registration process. Participants were instructed to refrain from eating, smoking, or chewing gum for at least 30 min prior to oral sample collection, and if applicable, wipe off any lipstick. Participants were instructed to drink one to two mouthfuls of water, then relax and gently rub cheeks to aid in saliva production. Participants deposited saliva (3 ml) into 50-ml sterile conical tubes. Samples were stored at 4 °C, batch shipped on ice, and processed within 24 h of collection at the central processing facility at the QEII Health Sciences Centre in Halifax where they were aliquoted and stored at − 80 °C until analysis.

### Frailty data

The FI was developed as previously described^36^. Briefly, using a standardized approach for the generation of a FI^40^, 38 health deficits including symptoms, signs, disabilities, disease, and laboratory measures related to physical, mental and self-reported health were included. The complete list of health deficits and coding procedure of each have been previously reported^36^. The index is expressed as a ratio of deficits present to the total number of deficits considered, with higher values representing a higher degree of frailty.

### Microbiome 16S rRNA gene sequencing analysis

Previously generated and processed 16S rRNA gene sequencing data from saliva samples was utilized for this study. DNA extraction, amplicon sequencing, and 16S rRNA gene sequencing data processing details have been previously published^28,37^. Briefly, the V4–V5 region of the 16S rRNA gene was amplified, and amplicon sequencing was performed on an Illumina Miseq. For processing, primers were removed, data was filtered, and amplicon sequence variants (ASVs) were produced using Deblur as a QIIME2 plugin^63–65^). The taxonomic classifications obtained previously were used to remove ASVs that were classified as Mitochondria or Chloroplasts and ASVs present in less than 5% of samples or at an abundance of less than 0.1% of the mean sample depth were removed from analyses. Taxonomy was assigned to the remaining ASVs using a scikit-learn classifier^66^ trained on the full-length 16S rRNA gene expanded Human Oral Microbiome Database (eHOMD; version 15.22)^39^ and samples were normalized by rarefying to the lowest read depth (2974 sequences) or conversion to robust centered log ratios (rCLR). Alpha and beta diversity were calculated for all samples using the scikit-bio^67^, scipy^68^ and deicode^69^ Python packages as well as the Phyloseq^70^ R package.

### Statistical analysis

Statistical analysis of participant characteristics and microbial diversity and composition was conducted using R Version 4.0.2. For the participant characteristics, categorical variables are presented as frequency (counts) and percentage (%), and continuous variables are presented as medians and interquartile ranges (IQR). For the statistical analysis of taxonomic data, alpha and beta diversity comparisons, principal coordinate analysis (PCoA) plots, and relative abundance plots were performed using the R packages vegan and ggplot2. Shannon Diversity, Richness (*observed number of ASVs*), Simpson’s Evenness, and Faith’s Phylogenetic Diversity on rarefied data were used to evaluate alpha diversity at the ASV level and Pearson correlation was used to assess the relationship between alpha diversity and age/frailty. Pearson correlation coefficients and p-values were calculated for each alpha diversity measure and age/frailty. Bray–Curtis dissimilarity and weighted UniFrac distance (on rarefied data) as well as Robust Aitchison’s Distance (Euclidean distance on rCLR data) were the metrics used to evaluate beta diversity at the ASV level. Beta diversity metrics were analysed using permutational multivariate analysis of variance PERMANOVA with 10,000 permutations using the adonis2 function within the vegan R package. An additional model was examined that adjusted for sex, smoking status, height, weight, vegetable consumption, and medication use. PCoA plots were used to visualize beta diversity metrics. An alpha value of 0.05 was chosen for determining significance of both alpha and beta diversity.

Genus abundance tables were used for differential abundance analysis of bacterial taxa. This was conducted using four different tools designed for differential abundance analysis: Corncob version 0.2.0^71^, ALDEx2 version 1.22.0^72^, MaAsLin2 version 1.4.0^73^, and ANCOM-II version 2.1^74^. For differential abundance testing, a prevalence cut-off filter was set to remove taxa found in fewer than 10% of samples. For ANCOM-II abundance tables were first processed using the function “feature_table_pre_process”, then the main “ANCOM” function, with a significance percentage cutoff of 80% for the w statistic. Corncob was run using “differentialTest” function with the wald test; ALDeX2 was run using the “aldex.glm” and “aldex.clr” functions with a total of 128 Monte Carlo samplings; MaAsLin2 was run using the function “maaslin2” with arcsine square root transformation and default parameters. With each of the above R packages, resulting p values were corrected for multiple hypothesis testing using the Benjamini and Hochberg algorithm and an alpha value of q = 0.1 was considered statistically significant. All differential abundance testing was also run with an additional model adjusted for sex, smoking status, standing height, body weight, diet (vegetable servings), and medication use. Taxa that remained significant in the fully adjusted model and identified by two or more of the differential abundance tools were considered noteworthy and further plotted to visualize the relationships. In addition, a sex-stratified analysis for all diversity and abundance tests was also conducted to explore variation in the microbiota across frailty/age groups in males and females.

### Supplementary Information

Supplementary Information.

## Data Availability

All sequence data has been uploaded to the European Nucleotide Archive and are available under the accession numbers PRJEB70783. Code used to analyze all data is available at https://github.com/vdeclercq/DECLERCQ_et_al_2023_Oral_Microbiome_Frailty. Metadata used in this project cannot be shared publicly because participant consent and ethical restrictions do not permit public sharing of the data. Data and biosamples from Atlantic PATH are available to researchers through a data access process. Additional information can be obtained by contacting info@atlanticpath.ca.
