# Supplementary material for: Oral microbial signatures associated with age and frailty in Canadian adults
Source: Sci Rep. 2024 Apr 27;14:9685. doi: 10.1038/s41598-024-60409-8 (PMC11055859; doi:10.1038/s41598-024-60409-8)
Supplement: Supplementary file 1 — Supplementary Information. [file 41598_2024_60409_MOESM1_ESM.pdf]

Oral microbial signatures associated with age and frailty in Canadian adults.

Vanessa DeClercq[1,2*], Robyn J. Wright[1], Jacob T Nearing[3], Morgan GI Langille[1,3]

[1] Department of Pharmacology, Dalhousie University, Nova Scotia, Canada
[2] Department of Community Health and Epidemiology, Dalhousie University, Nova Scotia, Canada
[3] Department of Microbiology and Immunology, Dalhousie University, Nova Scotia, Canada

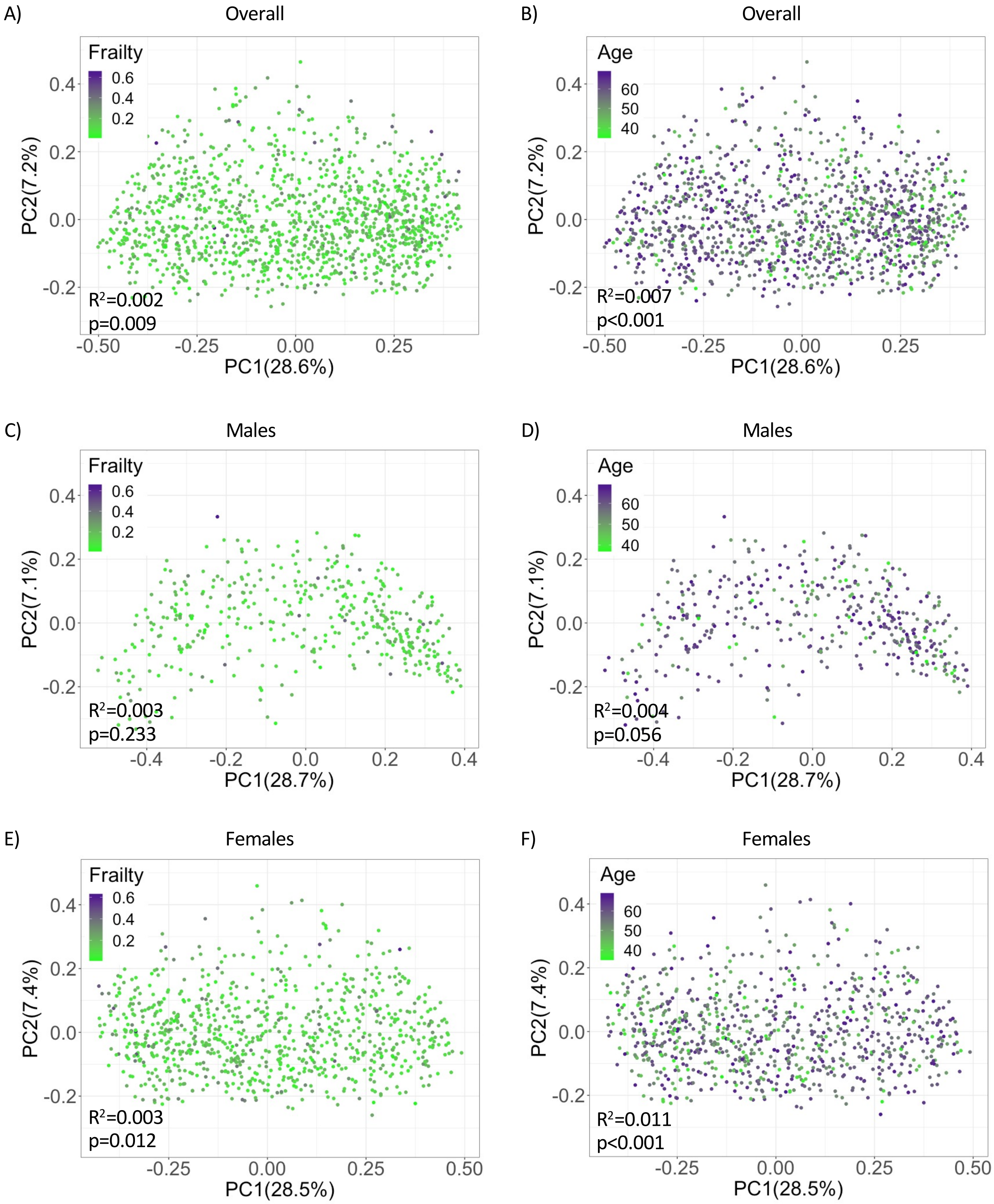

Supplemental Figure 1. **Beta diversity analyses among frailty and age groups are represented by Principal Coordinates Analysis plots based on Bray-Curtis.** (A and B) all participants, (C and D) male participants, (E and F) female participants. R^2 and p-values are the result of the unadjusted PERMANOVA test.

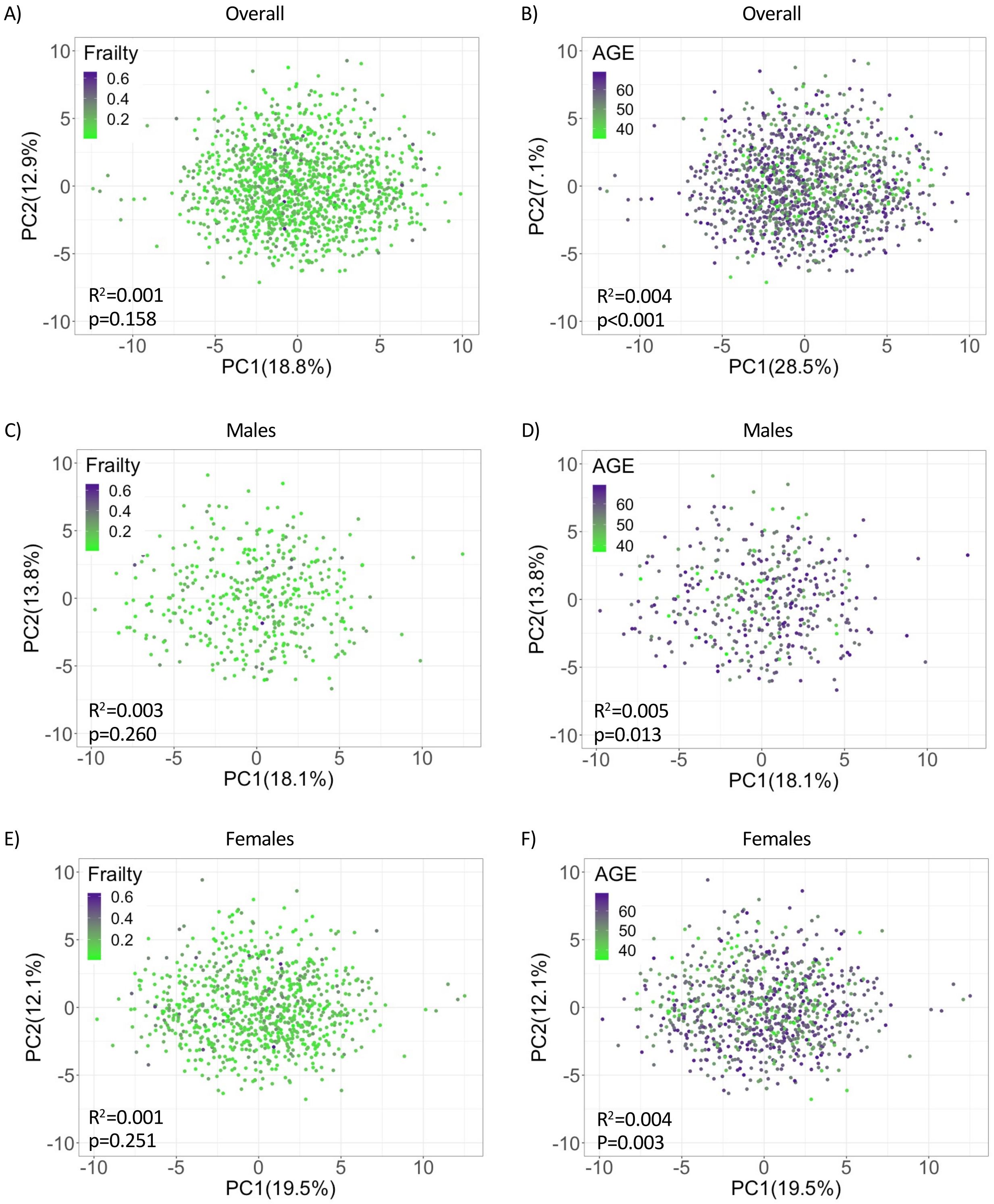

Supplemental Figure 2. **Beta diversity analyses among frailty and age groups are represented by Principal Coordinates Analysis plots based on Robust Aitchison.** (A and B) all participants, (C and D) male participants, (E and F) female participants. R^2 and p-values are the result of the unadjusted PERMANOVA test.

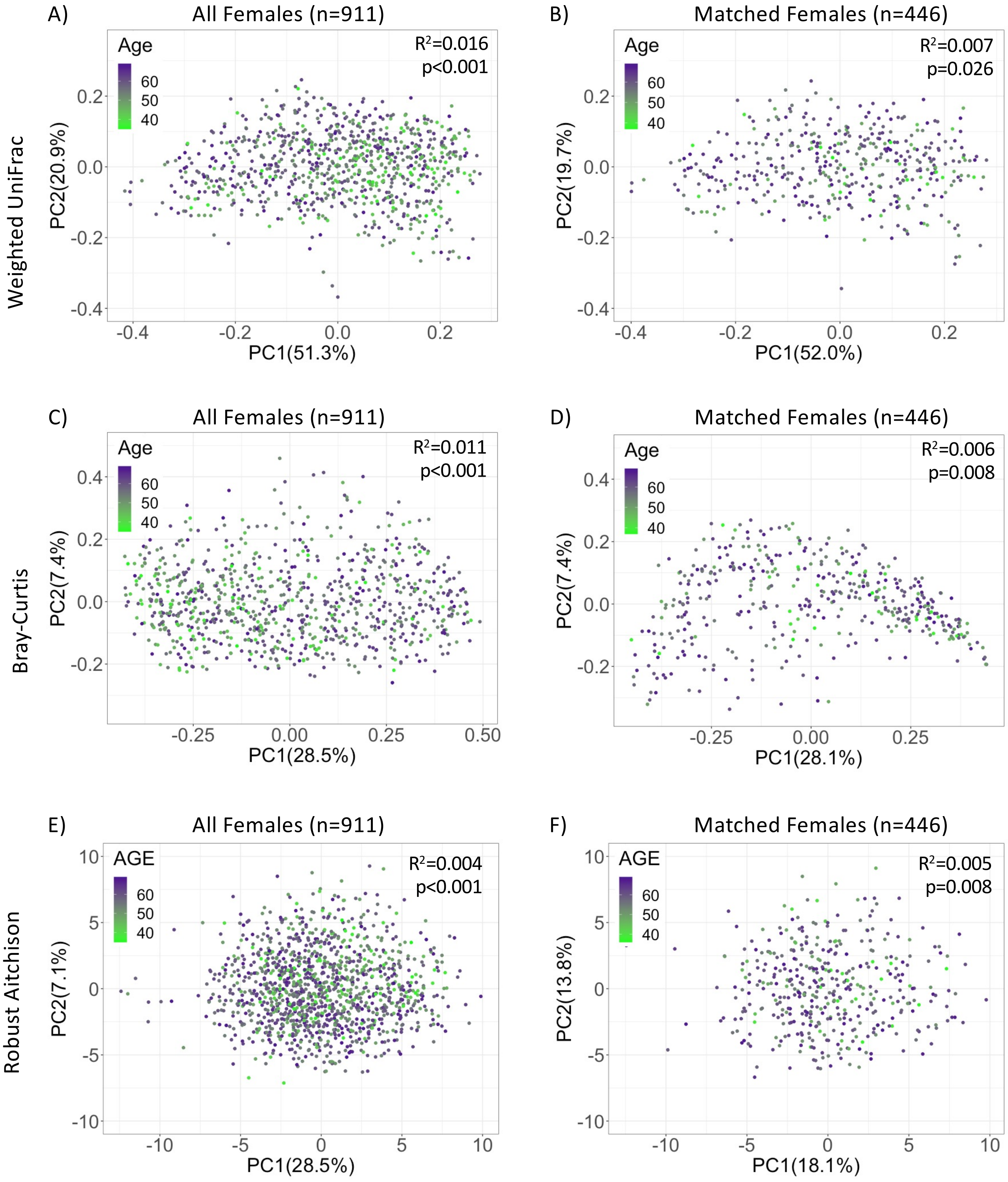

Supplemental Figure 3. Beta diversity analyses with chronological age is represented by Principal Coordinates Analysis plots. Weighted UniFrac (A and B) Bray-Curtis (C and D) and Robust Aitchison (E and F) for all females (A, C, and E) and for a subset of females (B, D, and F) matched to age (+/-2yrs) and sample size (n=446) of the male group. R^2 and p-values on plots are the result of the unadjusted PERMANOVA test.

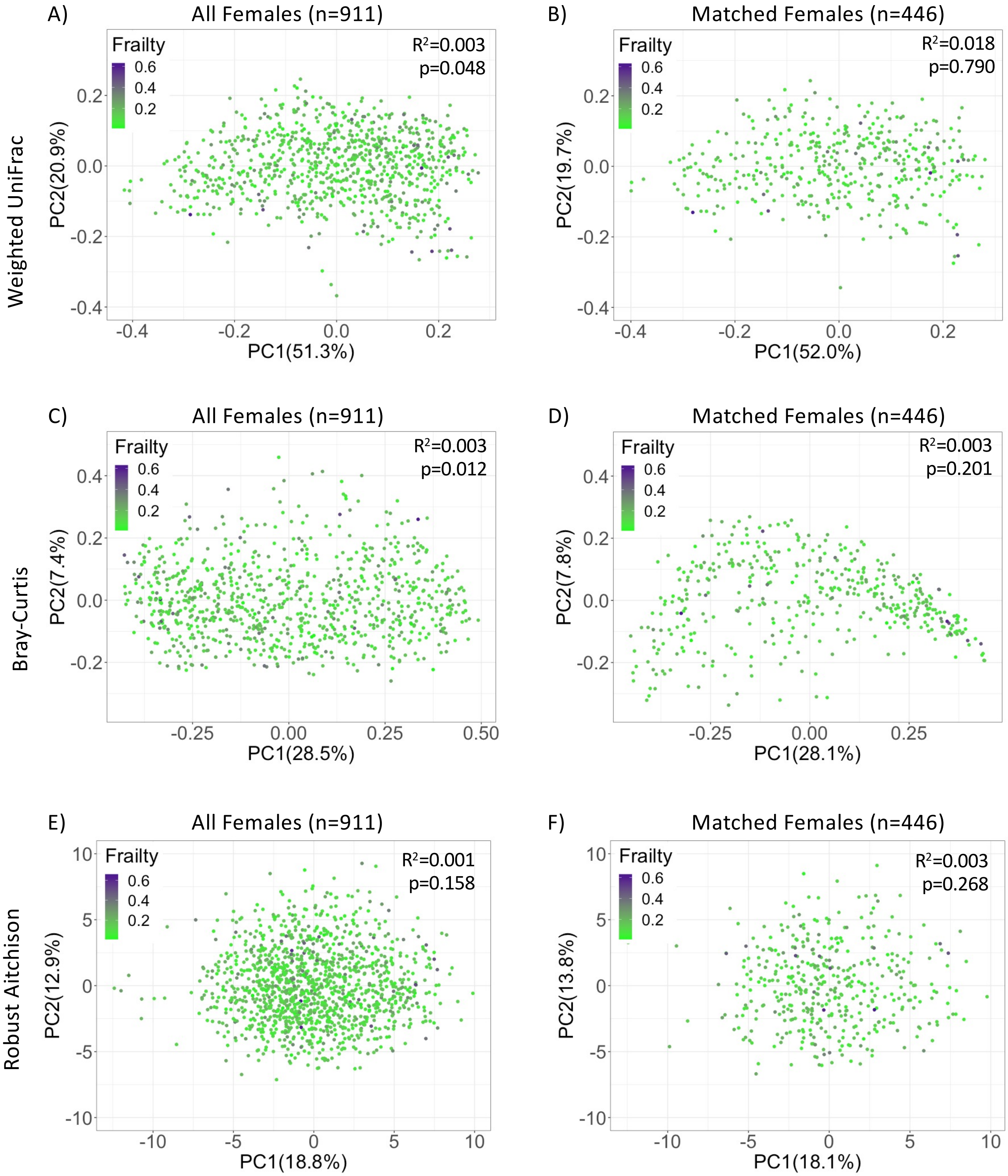

Supplemental Figure 4. Beta diversity analyses with frailty is represented by Principal Coordinates Analysis plots. Weighted UniFrac (A and B) Bray-Curtis (C and D) and Robust Aitchison (E and F) for all females (A,C, and E) and for a subset of females (B, D, F) matched to age (+/-2yrs) and sample size (n=446) of the male group. R^2 and p-values on plots are the result of the unadjusted PERMANOVA test.

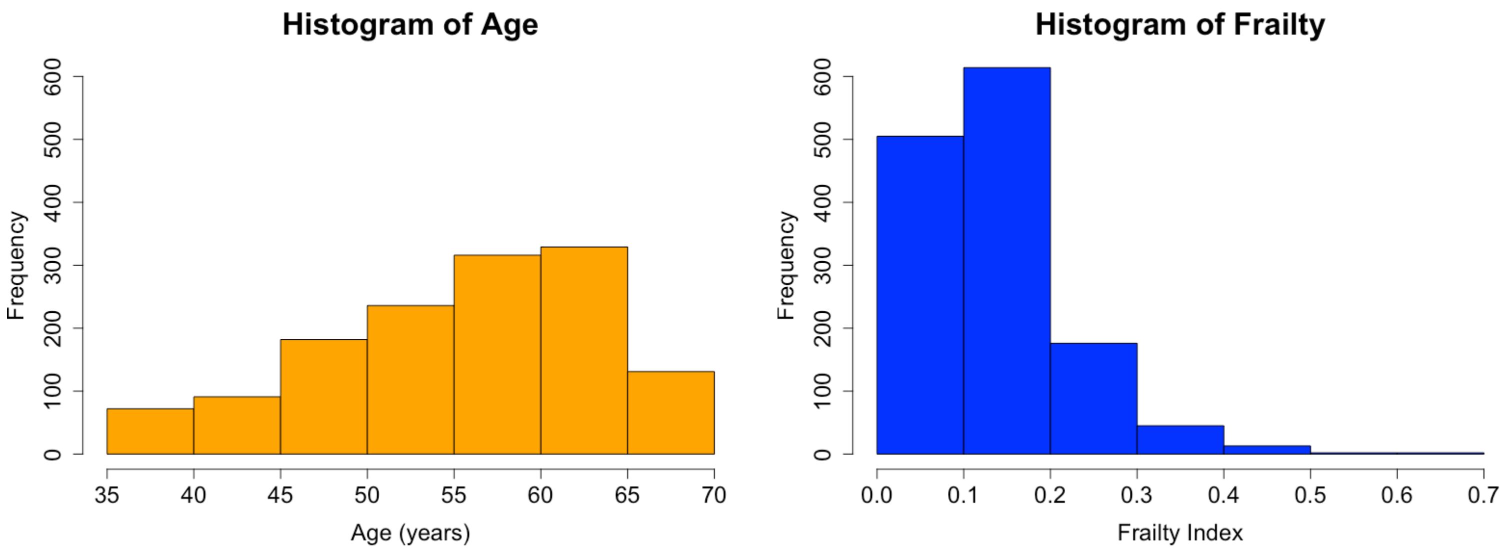

Supplemental Figure 5. Distribution of Age and FI among participants.

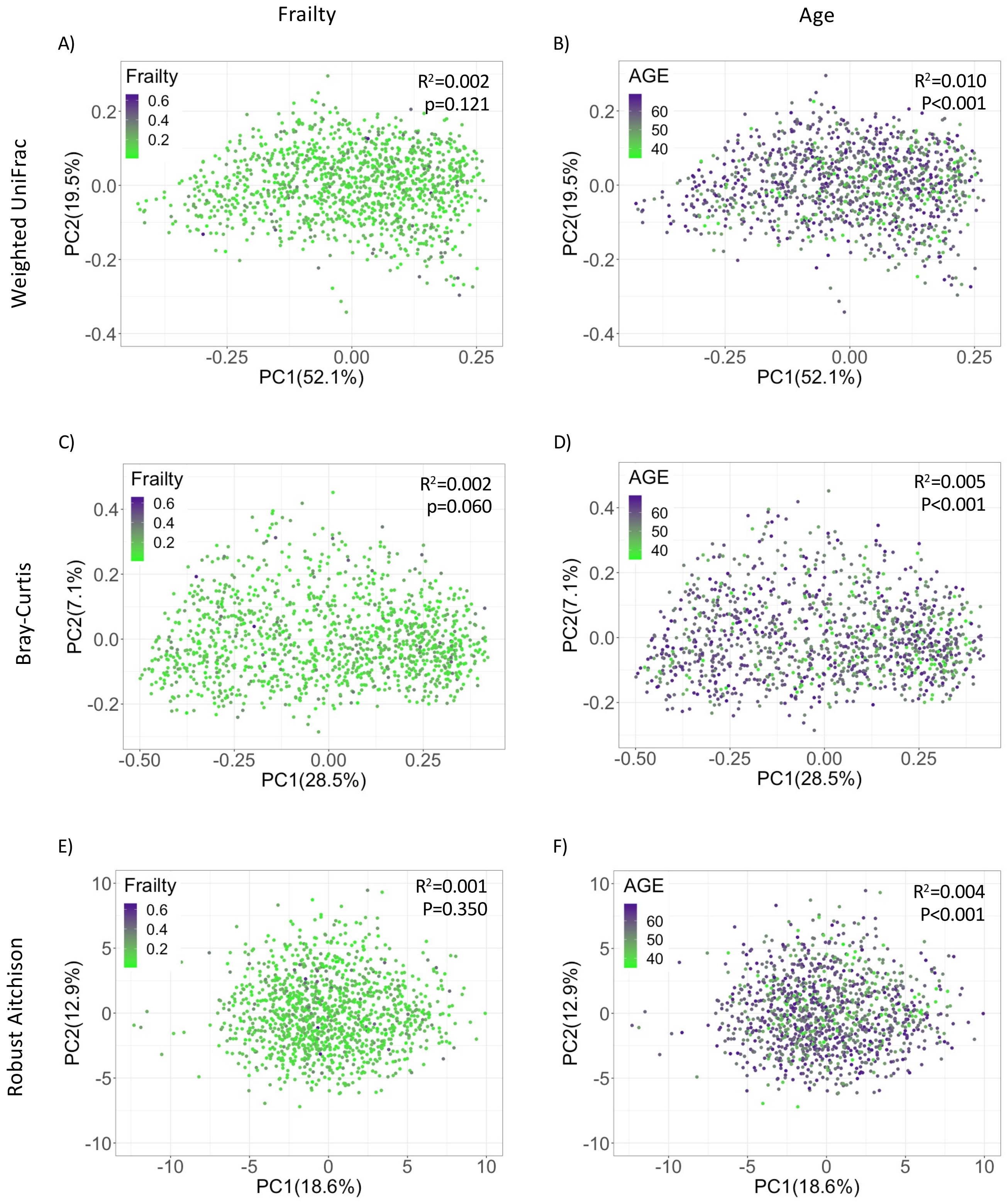

Supplemental Figure 6. Beta diversity in non-current daily are represented by Principal Coordinates Analysis plots based on (A, B) weighted UniFrac, (C, D) Bray-Curtis, and (E, F) Robust Aitchison. (A, C, and E) frailty, (B, D, and F) age. R^2 and p-values on the plots are the result of the unadjusted PERMANOVA test.

The adjusted PERMANOVA test with frailty were R^2=0.001, p=0.387 for weighted UniFrac, R^2=0.001, p=0.404 for Bray-Curtis dissimilarity, and R^2=0.001 p=0.667 for Robust Aitchison. The adjusted PERMANOVA test with age were for R^2=0.013, p<0.001 for weighted UniFrac, R^2=0.007, p<0.001 for Bray-Curtis dissimilarity, and R^2=0.004 p<0.001 for Robust Aitchison.
